# Inhibition of the cardiac fibroblast-enriched histone methyltransferase Dot1L prevents cardiac fibrosis and cardiac dysfunction

**DOI:** 10.1186/s13578-022-00877-5

**Published:** 2022-08-19

**Authors:** Jie Xu, Jinghuan Wang, Fen Long, Wen Zhong, Haibi Su, Zhenghua Su, Xinhua Liu

**Affiliations:** School of Pharmacy, Pharmacophenomics Laboratory, Human Phenome Institute, Fudan University, 825, Zhangheng Road, Pudong New District, Shanghai, 201203 People’s Republic of China

**Keywords:** Dot1L, Cardiac Fibrosis, Cardiac fibroblasts, Cardiac function, FoxO3a

## Abstract

**Background:**

Cardiac fibrosis is characterized by excessive extracellular matrix deposition that contributes to compromised cardiac function and potentially heart failure. Disruptor of telomeric silencing 1-like (Dot1L) is the catalytic enzyme required for histone H3K79 methylation which has been demonstrated to play a role in transcriptional activation. However, the functions of Dot1L in the process of cardiac fibrosis still remain unknown.

**Results:**

In the present study, we found that endogenous Dot1L is upregulated in cardiac fibroblasts (CFs) treated with angiotensin II (Ang II) or transforming growth factor (TGF)-β1, along with elevated extracellular matrix (ECM) such as fibronectin, collagen I and III. Silencing or inhibiting Dot1L mitigated Ang II-induced myofibroblast generation and fibrogenesis. We identified the transcription factor-forkhead box O (FoxO) 3a as a novel substrate of Dot1L, the transcriptional activating mark H3K79me3 level on the promoter of FoxO3a was increase in activated-CFs, and inhibition of Dot1L markedly decreased FoxO3a transcription accompanied by a significant decrease in the expression of fibrogenic gene. Knockdown of FoxO3a could alleviate ECM deposition induced by Ang II, on the contrary, overexpression FoxO3a resulting in CFs activation. Consistently, in vivo Dot1L ablation rescued myocardial ischemia-induced cardiac fibrosis and improved cardiac function.

**Conclusions:**

Our findings conclude that upregulation of Dot1L results in activation of the cardiac fibroblasts to promote profibrotic gene, eventually causes cardiac fibrosis. Pharmacological targeting for Dot1L might represent a promising therapeutic approach for the treatment of human cardiac fibrosis and other fibrotic diseases.

**Supplementary Information:**

The online version contains supplementary material available at 10.1186/s13578-022-00877-5.

## Introduction

Myocardial fibrosis (that is, excessive deposition of scar tissue) comes with numerous forms of heart disease such as myocardial infarction (MI), cardiomyopathy [1], myocarditis [2], and infiltrative diseases [3], and the following heart failure is the leading cause of death in the world [4, 5]. Myocardial fibrosis manifests as excessive deposition of extracellular matrix (ECM), along with the synthesis and arrangement of series of ECM proteins disordering, significantly decreased cardiac output, which eventually contributes to the progression of heart failure. Despite substantial improvements in therapeutic strategies, there are few effective therapeutic approaches for suppressing the development of myocardial fibrosis. Thus, therapies and strategies to reduce myocardial fibrotic changes are extremely important in treating cardiovascular disease.

The tissue-resident cardiac fibroblast (CFs) is the predominant cell to mediate progressive cardiac fibrosis. Under pathological stimulation, they become activated and then differentiated into the myofibroblast, which is known as fibroblast-to-myofibroblast transition (FMT) [6, 7]. Numerous studies suggested that levels of circulating hormones such as angiotensin II (Ang II) and fibrogenic cytokines transforming growth factor are contributed to the activation of fibroblasts [8, 9]. The myofibroblasts are the most important effector cells in the process of myocardial fibrosis [10] and also responsible for secreting numerous cytokines, growth factors, and ECM that eventually resulted in cardiac fibrosis [11]. Thus, inhibition of myofibroblast conversion or their activity would be an attractive therapeutic strategy in adult fibrotic disease states, including heart failure [12].

In recent years, the breakout of epigenetics gradually penetrates into a variety of biological system [13]. Multiple epigenetic mechanisms are highly interdependent and closely associated with gene expression, including histone modifications, DNA methylation and regulatory noncoding RNAs [14]. Large numbers of transcription factors and signal transduction molecules are dynamic regulated by these epigenetic mechanisms through chromatin remodeling or other modifications on gene level [15]. Emerging data suggest that these epigenetic modifications also impact on the development of cardiac fibrosis [16, 17]. Dot1L (disruptor of telomeric silencing 1-like) is the only known H3K79 methyltransferase [18], and H3K79 methylation is mainly considered transcriptional activating when highly abundant. Dot1L was found to play an essential role in transcriptional elongation, DNA repair and cell cycle regulation [19, 20], making it an attractive therapeutic target for acute leukemia [21]. However, the functional role of Dot1L in the induction of cardiac fibrosis has still been vague.

Therefore, this study first provided insight into the functional role and mechanisms of Dot1L in cardiac fibrosis. Using combinative in vitro and in vivo studies, we demonstrated that Dot1L is a critical component of pro-fibrotic signaling in the heart, and determined the therapeutic potential of specific inhibitor EPZ5676 in cardiac fibrosis of post-MI. Furthermore, transcription factor forkhead box O (FoxO) 3a was identified as a specific and effective target for Dot1L in activation of the cardiac fibroblasts, and we concluded that inhibition of the cardiac fibroblast-enriched histone methyltransferase Dot1L alleviates cardiac fibrosis and cardiac dysfunction.

## Results

### Dot1L is upregulated in TGF-β1-induced adult rat CFs and fibrotic mouse hearts

**Fig. 1:**
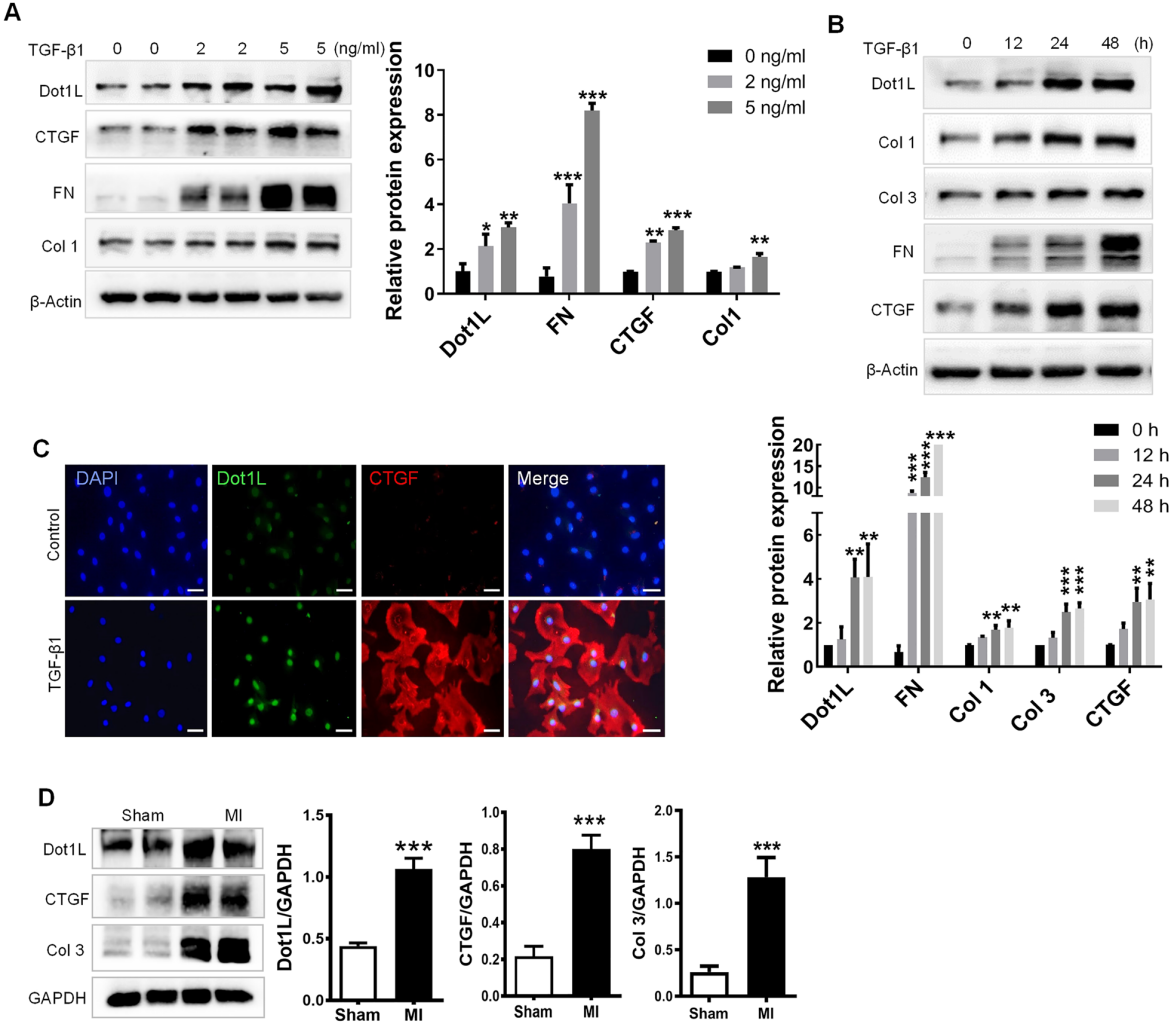
Dot1L is upregulated in TGF-β1-induced adult rat CFs and fibrotic mice hearts. **A** Dot1L, Col 1, FN, and CTGF protein expression level in cardiac fibroblasts (CFs) stimulated by different doses of TGF-β1 (0, 2, and 5 ng/mL) for 48 h. **B** Col 1, CTGF, Col 3, FN, and Dot1L protein expression level in cardiac fibroblasts stimulated by TGF-β1 (5 ng/mL) for the dedicated time (0, 12, 24, and 48 h). The data are presented as mean ± SD. Student’s t-test, ^*^p < 0.05, ^**^p < 0.01, and ^***^p < 0.001 versus control group, each acquired from three individual experiments. **C** Representative images of immunofluorescence staining of Dot1L as well as CTGF in CFs stimulated by 5 ng/mL TGF-β1 for 48 h. **D** Dot1L, Col 3 and CTGF protein expression around infarct area were measured in mice undergoing 2 weeks of left descending anterior coronary artery (LAD) ligation. The data are presented as mean ± SD. Student’s t-test, ^***^p < 0.001 versus Sham group, n = 6/group

TGF-β1 can drive myofibroblast activation, as Fig. 1A showed, markers of fibrosis such as Collagen I (Col 1), fibronectin (FN) and connective tissue growth factor (CTGF) were abundantly expressed in activated CFs by different concentration of TGF-β1, as well as Dot1L expression. Besides, an increased expression of Dot1L was presented at different time after stimulation with TGF-β1, along with expressions of Col 1, Col 3, FN and CTGF (Fig. 1B). Furthermore, immunofluorescence double staining showed that the expressions of Dot1L and CTGF were increased in TGF-β1-induced CFs (Fig. 1C). Thus, 5 ng/mL TGF-β1 was applied to activate adult rat CFs for 48 h in subsequent experiments.

To further explore whether Dot1L level was increased in response to cardiac fibrosis in vivo, cardiac fibrosis post-MI was induced by permanent ligation of the left ascending artery (LAD) in mice. Consistently, after 2 weeks of LAD ligation, MI model mice exhibited obvious upregulation of Dot1L in comparison with sham mice, along with notably increase of protein levels of the fibrosis markers Col 3 and CTGF (Fig. 1). These results suggest that Dot1L is upregulated during MI and may participate in cardiac fibrosis.

### Dot1L ablation attenuates TGF-β1-induced ECM deposition in adult rat CFs

**Fig. 2:**
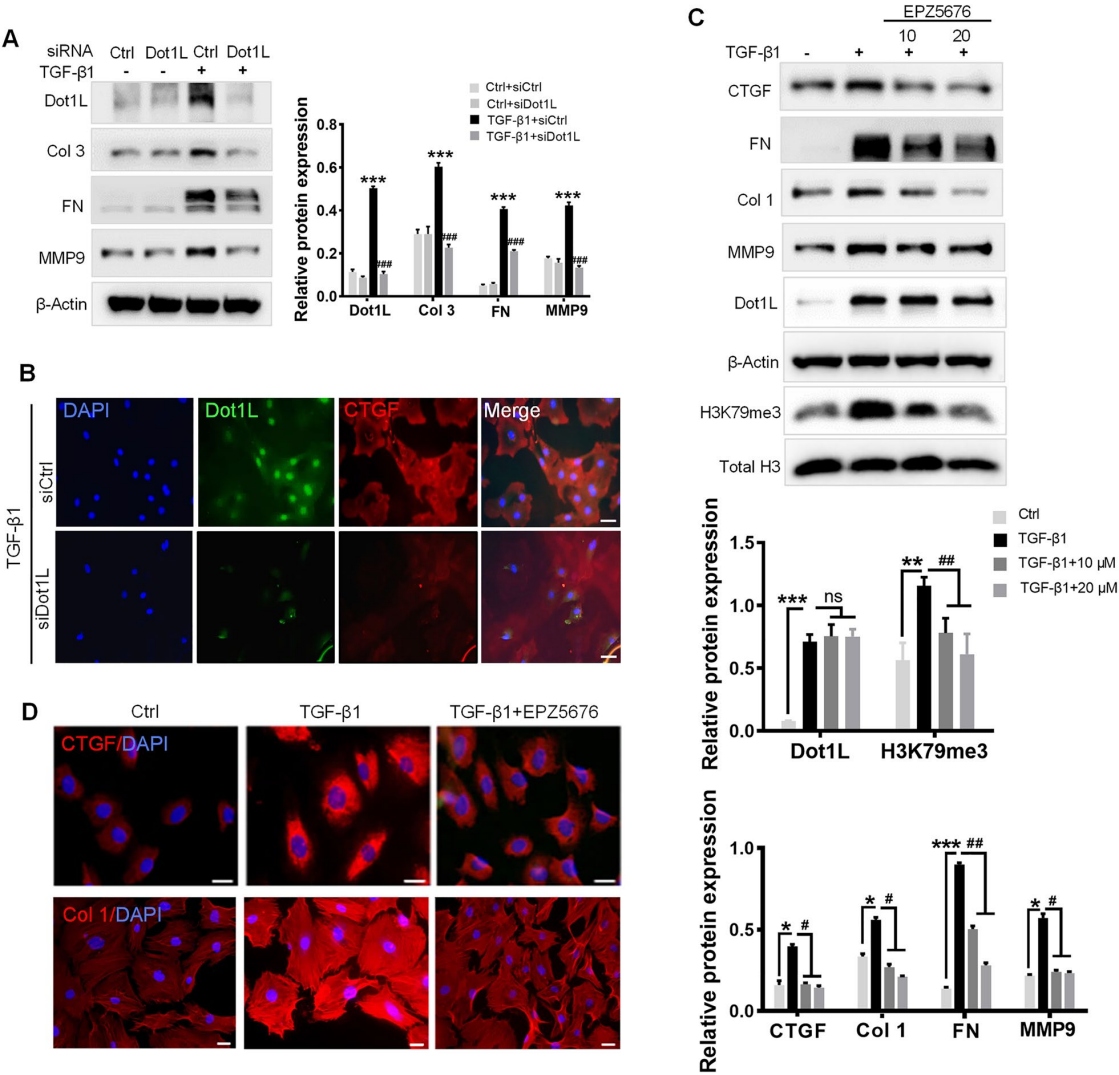
Knockdown or inhibition of Dot1L attenuates TGF-β1-induced ECM deposition in adult rat CFs. **A**, **B** Knockdown of Dot1L attenuates ECM deposition in TGF-β1 treated CFs. Dot1L siRNA and control siRNA were transfected into CFs, followed by 5 ng/mL TGF-β1 stimulation for 48 h. The protein expressions of Dot1L, Col 3, FN and MMP9 were analyzed by western blot (**A**); Representative images of immunofluorescence staining of Dot1L and CTGF (**B**). **C**, **D** EPZ5676 attenuates ECM deposition in TGF-β1-treated CFs. CFs were pretreated with 10 and 20 μmol/L EPZ5676 for 4 h and then co-incubated with 5 ng/mL TGF-β1 for 48 h. H3K79me3, Dot1L, CTGF, Col 1, MMP9 and FN were analyzed by western blot (**C**); Representative images of immunofluoresence staining of CTGF or Col 1 were shown in 20 μmol/L EPZ5676-treated CFs (**D**). All data are presented as mean ± SD. ANOVA, ^*^p < 0.05, ^**^p < 0.01, ^***^p < 0.001 versus Ctrl or Ctrl + siCtrl, ^#^*p* < 0.05, ^##^*p* < 0.01, ^###^*p* < 0.001 versus TGF-β1 or TGF-β1 + siCtrl, each acquired from three individual experiments

We next explored the direct link between Dot1L upregulation and fibrosis in cultured adult rat CFs. Firstly, to verify whether silencing Dot1L would prevent fibrosis in adult rat CFs, we used siRNA to knock down Dot1L expression (Fig. 2A). Consistently, silencing Dot1L prevented the TGF-β1-induced the increase in the expression of fibrosis-related markers, such as FN, Col 3, and matrix metalloproteinase (MMP) 9 (Fig. 2A). In addition, immunofluorescence double staining also confirmed silencing Dot1L decreased CTGF expression (Fig. 2B).

Small molecule Dot1L inhibitor such as EPZ5676 can inhibit methylation effect of Dot1L on H3K79, as Dot1L is the only methyltransferase acting on histone H3K79. We found that EPZ5676 co-treatment could decrease H3K79me3 expression and the synthesis of TGF-β1-induced CTGF, FN, Col 1, and MMP9 to below baseline levels (Fig. 2C). Immunofluorescence staining also confirmed EPZ5676 treatment exhibited decreased CTGF and Col 1 expressions upon TGF-β1 (Fig. 2D). These results demonstrate that knocking down or inhibiting Dot1L could suppress TGF-β1-induced ECM deposition in vitro.

### Dot1L ablation attenuates Ang II-induced fibrosis in neonatal rat CFs (NRCFs)

**Fig. 3:**
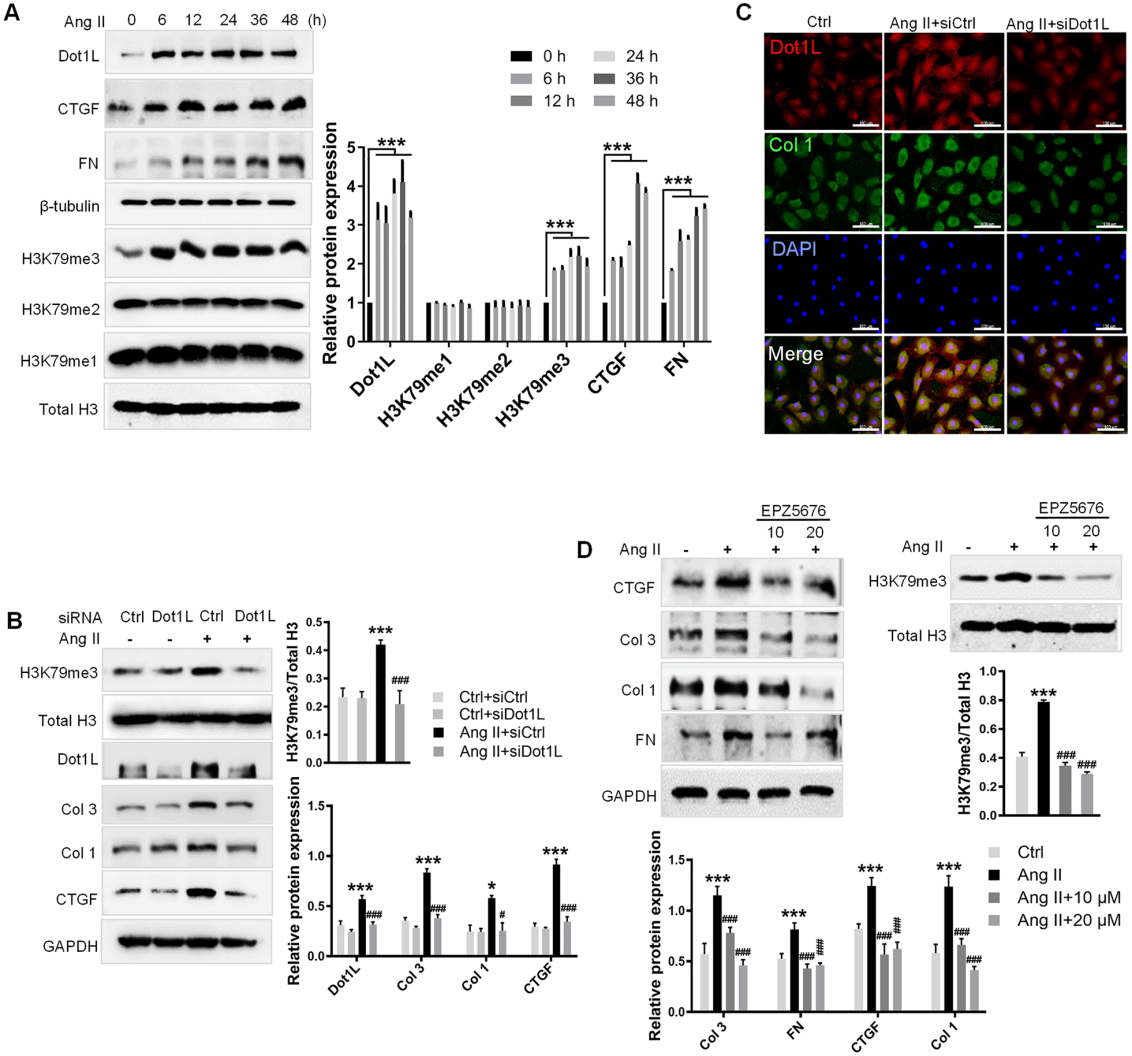
Knockdown or inhibition of Dot1L attenuates Ang II-induced fibrosis in neonatal rat CFs (NRCFs). **A** Dot1L, CTGF, FN, and H3K79me3/2/1 protein expression level in NRCFs stimulated by Ang II (2 μmol/L) for different time (0, 6, 12, 24, 36, and 48 h). **B**, **C** Knockdown of Dot1L attenuates ECM deposition in Ang II-treated NRCFs. Dot1L siRNA and control siRNA were transfected into NRCFs, followed by Ang II stimulation for 48 h. The protein expressions of Dot1L, Col 1, Col 3, CTGF, and H3K79me3 were analyzed by western blot (**B**); Representative images of immunofluorescence staining of Dot1L and Col 1 (**C**). **D** EPZ5676 attenuates ECM deposition in Ang II-treated NRCFs. NRCFs were pretreated with 10 and 20 μmol/L EPZ5676 for 4 h and then co-incubated with 2 μmol/L Ang II for 48 h. CTGF, Col 3, Col 1, FN, and H3K79me3 were analyzed by western blot. All data are presented as mean ± SD. ANOVA, ^*^p < 0.05, ^***^p < 0.001 versus Ctrl or Ctrl + siCtrl, ^#^*p* < 0.05, ^###^*p* < 0.001 versus Ang II or Ang II + siCtrl, each acquired from three individual experiments

Ang II also plays an important role in myocardial remodeling after MI [22, 23], we applied Ang II-induced NRCFs and analyzed the relative levels of Dot1L and collagen synthesis. We performed western blot to examine Dot1L expression and markers of fibrosis, which was up-regulated by Ang II (Fig. 3A). In addition to the dynamic change in Dot1L expression, we also observed increased deposition of H3K79me3 with no obvious change in H3K79me2/me1 expression, providing an assumption that Dot1L played an important role in cardiac fibrosis via its tri-methylation on H3K79 (Fig. 3A). Furthermore, we treated NRCFs with Dot1L siRNA to determine the effect of Dot1L on Ang II-induced fibrosis, and found silencing Dot1L significantly decreased the expression of CTGF, Col 1 and Col 3 in NRCFs (Fig. 3B). Consistent with a reduced expression of Dot1L expression, silencing Dot1L exhibited a decrease in H3K79me3 abundance relative to total histone H3 levels (Fig. 3B). Similarly, immunofluorescence assay further confirmed that Ang II enhanced the co-expression between Dot1L and Col 1, while silencing of Dot1L decreased Col 1 expression (Fig. 3C). Pretreating the Ang II-stimulated fibroblasts with EPZ5676 suppressed fibrosis-related markers, such as FN, CTGF, Col 1, and Col 3 expression (Fig. 3D). Consistently, EPZ5676 exhibited a decrease in H3K79me3 abundance relative to total histone H3 levels in activated neonatal rat CFs (Fig. 3D). Together, we had similar findings in Ang II-stimulated CFs from neonatal rat, where Dot1L levels were markedly increased, and ablation of Dot1L inhibited fibroblast activation. The change in Dot1L ablation was consistent with TGF-β1-stimulated CFs from adult rat. These observations support that Dot1L contributes to cardiac fibrosis.

### Inhibiting Dot1L prevents cardiac fibrosis and dysfunction during MI

**Fig. 4:**
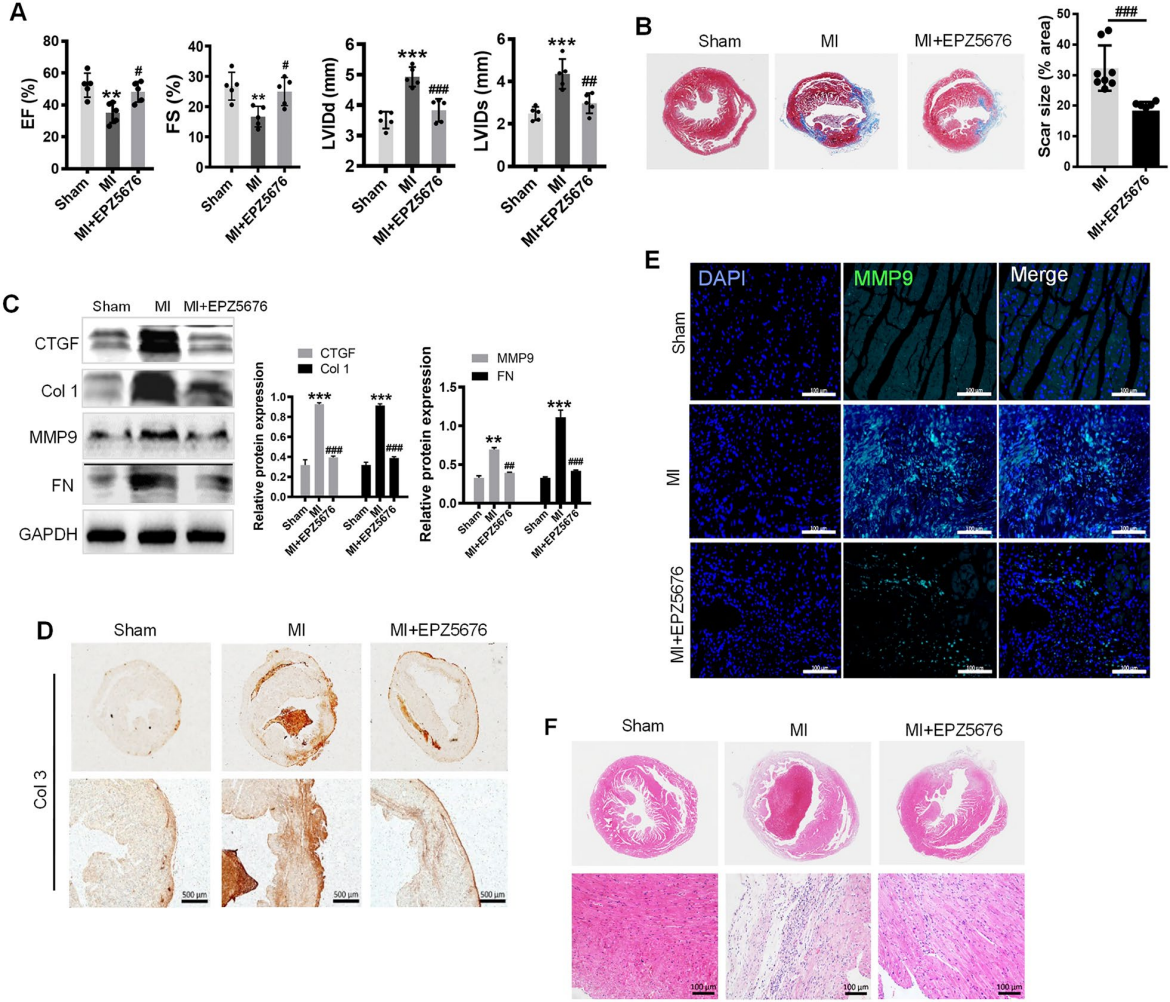
Inhibiting Dot1L prevents cardiac fibrosis and improves cardiac function during MI. EPZ5676 at a dose of 20 mg/kg/day was administered to mice undergoing LAD ligation by intraperitoneal injection for 14 days. **A** The echocardiographic parameters at the 14th day post-MI were statistically analyzed and shown in the graphs, including EF%, FS%, LVIDs and LVIDd. **B** Masson’s trichrome staining of cross sections of whole heart and Scar size were analyzed. **C** Western blot was performed to analyze the expression of CTGF, Col 1, MMP9 and FN. **D** Representative images of immunohistochemistry staining of Col 3. **E** Representative images of immunofluorescence staining of MMP9. **F** Representative images of H&E staining at magnification of 50 X and 200 X respectively. Data are represented as means ± SD. ANOVA, ^**^p < 0.01, ^***^p < 0.001 versus Sham group, ^#^*p* < 0.05, ^##^*p* < 0.01, ^###^*p* < 0.001 versus MI, n = 5–8/per group

To explore the role of Dot1L during cardiac fibrosis post LAD-induced MI, we conducted a LAD procedure followed by intraperitoneal injection of EPZ5676 (20 mg/kg/day) from 1 day after LAD for 2 weeks. To assess cardiac function, we performed echocardiographic on day 14 post-LAD. Echocardiographic showed that comparing with the sham group, LAD treatment worsened both left ventricular (LV) function and structure, as indicated by expanded LV dimension and weakened LV systolic function. EPZ5676 treatment protected against the LAD-induced cardiac dysfunction (Fig. 4A). We next investigated whether EPZ5676 treatment of mice for 2 weeks could ameliorate the LAD-induced cardiac fibrosis, as deposition of fibrotic tissue was main cause of unfavourable heart remodeling in MI. Therefore, we evaluated the heart fibrosis degree by Masson’s trichrome staining, obviously, EPZ5676 treatment effectively alleviated cardiac fibrosis reflected with marked reduction in fibrosis area and degree (Fig. 4B). After a 2-week treatment, EPZ5676 significantly alleviated the protein levels of the cardiac fibrosis markers Col 1, CTGF, MMP9 and FN (Fig. 4C). Consistently, Col 3 expression were notably increased in MI mice compared with all other groups by immunohistochemistry (IHC) analysis (Fig. 4D). Furthermore, immunofluorescence analysis of sections of hearts further revealed that MMP9 protein expression was downregulated while treated with EPZ5676 (Fig. 4E). Compared to clear transverse striations in sham group shown by H&E staining, the myocardial cells in mice after LAD ligation were irregular shape and the transverse striations were blurry and broken. Whereas, in EPZ5676-treated mice after LAD ligation, myocardial cells were neatly arranged with decreased degree of fibroplasias (Fig. 4F). Taken together, the results confirm the pathogenesis of cardiac fibrosis in MI mice and inhibiting Dot1L attenuates the pathological remodeling and improves cardiac function.

### Knocking down Dot1L alleviates cardiac fibrosis and improves cardiac function

**Fig. 5:**
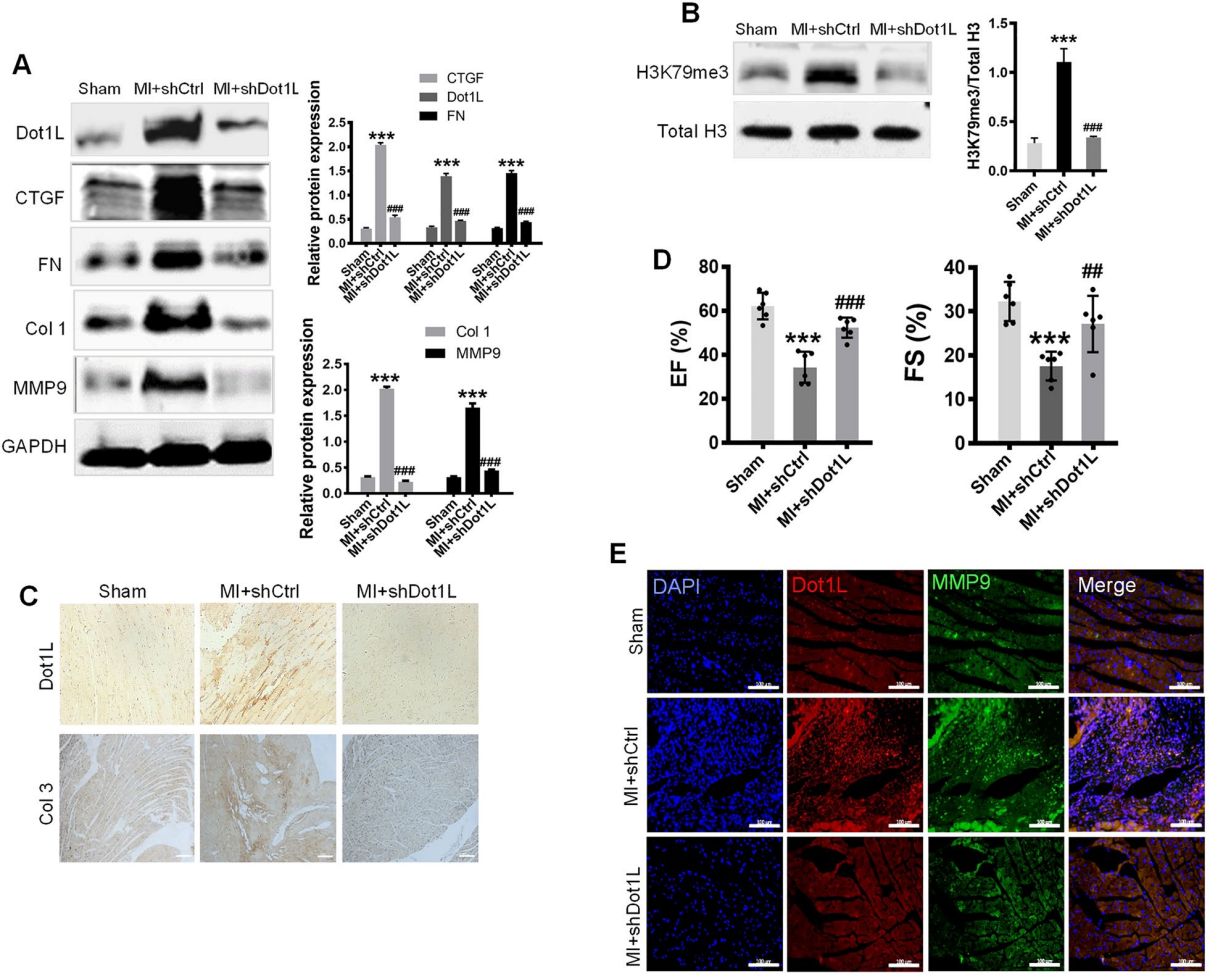
Knockdown of Dot1L alleviates cardiac fibrosis and improves cardiac function during MI. Lentivirus carrying Dot1L shRNA (shDot1L) or Control shRNA (shCtrl) was injected into myocardium of mice undergoing LAD ligation for 14 days to specially knock down the cardiac Dot1L. **A** The expressions of Dot1L, CTGF, FN, Col 1, and MMP9 in MI mice were analyzed by western blot. **B** The level of H3K79me3 was analyzed by western blot. **C** Representative images of immunohistochemistry staining of Dot1L and Col 3 of cross sections of whole heart from MI mice. **D** Cardiac functions were measured by echocardiography and EF%, FS% were also statistically analyzed. **E** Representative images of immunofluorescence double staining of Dot1L and MMP9 of cross sections of whole heart. All data are represented as means ± SD. ANOVA, ^***^p < 0.001 versus Sham group, ^##^*p* < 0.01, ^###^*p* < 0.001 versus MI treated with shCtrl, n = 6/per group

To elucidate the important role of Dot1L in cardiac fibrosis after injury, we generated a lentivirus carrying Dot1L shRNA. After the LAD ligation, knockdown of cardiac Dot1L was successfully determined by injection of lentivirus carrying Dot1L shRNA into myocardium at several positions surrounding the infarct border zone. Firstly, we verified the decrease of Dot1L protein expression in heart (Fig. 5A). In accordance with the findings above, the concomitant expressions of CTGF, FN, MMP9, and Col 1 were dramatically increased in the MI mice compared with the sham group. However, MI mice injected with Dot1L shRNA showed great reduction in those fibrosis markers in comparison with the MI mice (Fig. 5A), illustrating a pivotal role of Dot1L in cardiac myofibroblast activation in vivo. Consistent with a reduced expression of Dot1L, H3K79me3 abundance relative to total histone H3 level exhibited a decrease (Fig. 5B). IHC further confirmed the knockdown of Dot1L attenuated the expressions of Dot1L and Col 3 (Fig. 5C). To evaluate whether cardiac function was influenced by knockdown of Dot1L, echocardiograph was performed. MI mice injected with Dot1L shRNA exhibited remarkable improvement in ejection fraction (EF) and fractional shortening (FS) compared with MI mice (Fig. 5D). Besides, immunofluorescence was performed to check synchronous change between Dot1L and MMP9, which turned out to be consistent with the western blot results (Fig. 5E). In summary, the results suggest that knockdown of Dot1L alleviates cardiac fibrosis and ameliorates cardiac function after MI.

### Dot1L^+/-^ mice present the cardio-protective effect during MI

**Fig. 6:**
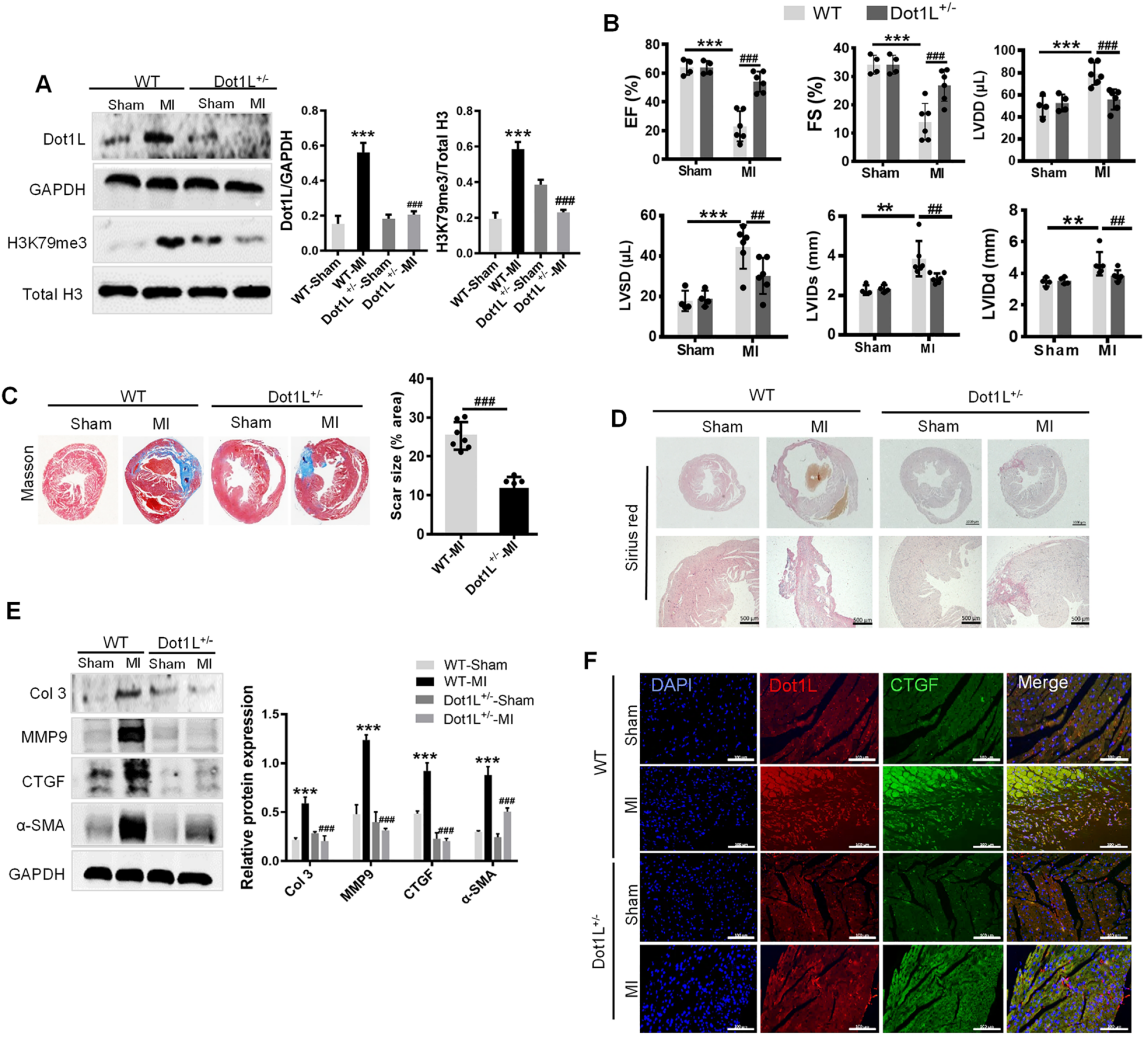
Dot1L heterozygote (Dot1L^+/-^) mice present the cardio-protective effect during MI. Dot1L^+/-^ and WT mice were undergone LAD ligation for 14 days to determine the functions of Dot1L in cardiac fibrosis. **A** The expressions of Dot1L and H3K79me3 in myocardium from Dot1L^+/-^ and WT mice at 14 days post-MI were examined through western blot. **B** The echocardiographic parameters at the 14th day post-MI in Dot1L^+/-^ and WT mice undergoing LAD ligation. **C** Representative images of Masson trichrome staining in Dot1L^+/-^ and WT mice undergoing LAD ligation for 14 days and scar size were analyzed. **D** Representative images of picrosirius red staining in Dot1L^+/-^ and WT mice undergoing LAD ligation. **E** The protein expressions of Col 3, MMP9, CTGF and α-SMA in Dot1L^+/-^ and WT mice undergoing LAD ligation were analyzed by western blot. **F** Representative images of immunofluorescence double staining of Dot1L and CTGF of cross sections of whole heart. Data are represented as means ± SD. ANOVA, ^**^p < 0.01, ^***^p < 0.001 versus WT Sham group, ^##^*p* < 0.01, ^###^*p* < 0.001 versus WT-MI group, n = 4–7/per group

Next, we generated mice with a whole-body knocking down to determine Dot1L functions in cardiac fibrosis. Dot1L heterozygotes (Dot1L^+/-^) were studied because homozygotes are not viable. The relative decrease of Dot1L protein level in heart was verified (Fig. 6A). Consistent with a reduced expression of Dot1L, Dot1L^+/-^ mice exhibited a decrease in H3K79me3 abundance relative to total histone H3 levels (Fig. 6A). Dot1L^±^ and WT mice were performed with LAD ligation. After 2 weeks, post-infarct ventricular remodeling in WT and Dot1L^+/-^ mice was assessed with echocardiography. WT and Dot1L^±^ hearts had comparable chamber dimensions and ventricular function before LAD ligation, but after 2 weeks post-MI, Dot1L^±^ animals had an improved LV function as reflected by increased EF and FS (Fig. 6B). Then, Masson trichrome staining and Sirius red staining were used to determine the extent of cardiac fibrosis, showing that Dot1L^±^ mice with LAD ligation exhibited relieved cardiac fibrosis compared with WT mice with LAD ligation (Fig. 6C, D). As expected, Col 3, MMP9, CTGF, and α-smooth muscle actin (α-SMA) expressions in Dot1L^±^ mice after ligation were significant reduced compared with WT mice receiving ligation, where there was nearly no comparable change between Dot1L^±^ mice and WT mice before ligation (Fig. 6E). Further, immunofluorescence double staining analysis of sections of hearts revealed that Dot1L^±^ mice had a decreased CTGF protein expression (Fig. 6F). In a word, these findings indicate Dot1L regulated cardiac remodeling after injury.

### Dot1L expression is mediated by p-Smad2/3 in Ang II-induced CFs

The Smad2/3 signaling pathway was activated in cardiac fibrosis, we examined whether Smad2/3 activation up-regulated Dot1L expression. We found that Ang II stimulated phosphorylation of Smad2/3 in CFs (Additional file1: Fig S1A**)**. To further validate the Smad2/3 regulatory role on Dot1L, Smad2/3 was knocked down by siRNA. The result showed that depletion of Smad2/3 led to a decrease in Smad2/3 and Dot1L protein level, along with reduced fibrosis markers such as CTGF and Col 3 (Additional file1: Fig S1B**)**. Meanwhile, Sis, an inhibitor of Smad3, was also proved to decrease Dot1L and fibrosis markers expression in a dose-dependent manner, showing that reduction of Smad2/3 activity can reverse increased expression of Dot1L (Additional file1: Fig S1C**).** These results suggest that upregulation of Dot1L in Ang II-induced cells is mediated by Smad3-dependent mechanism.

### Dot1L directly binding to the promoter regions of FoxO3a activates CFs

**Fig. 7:**
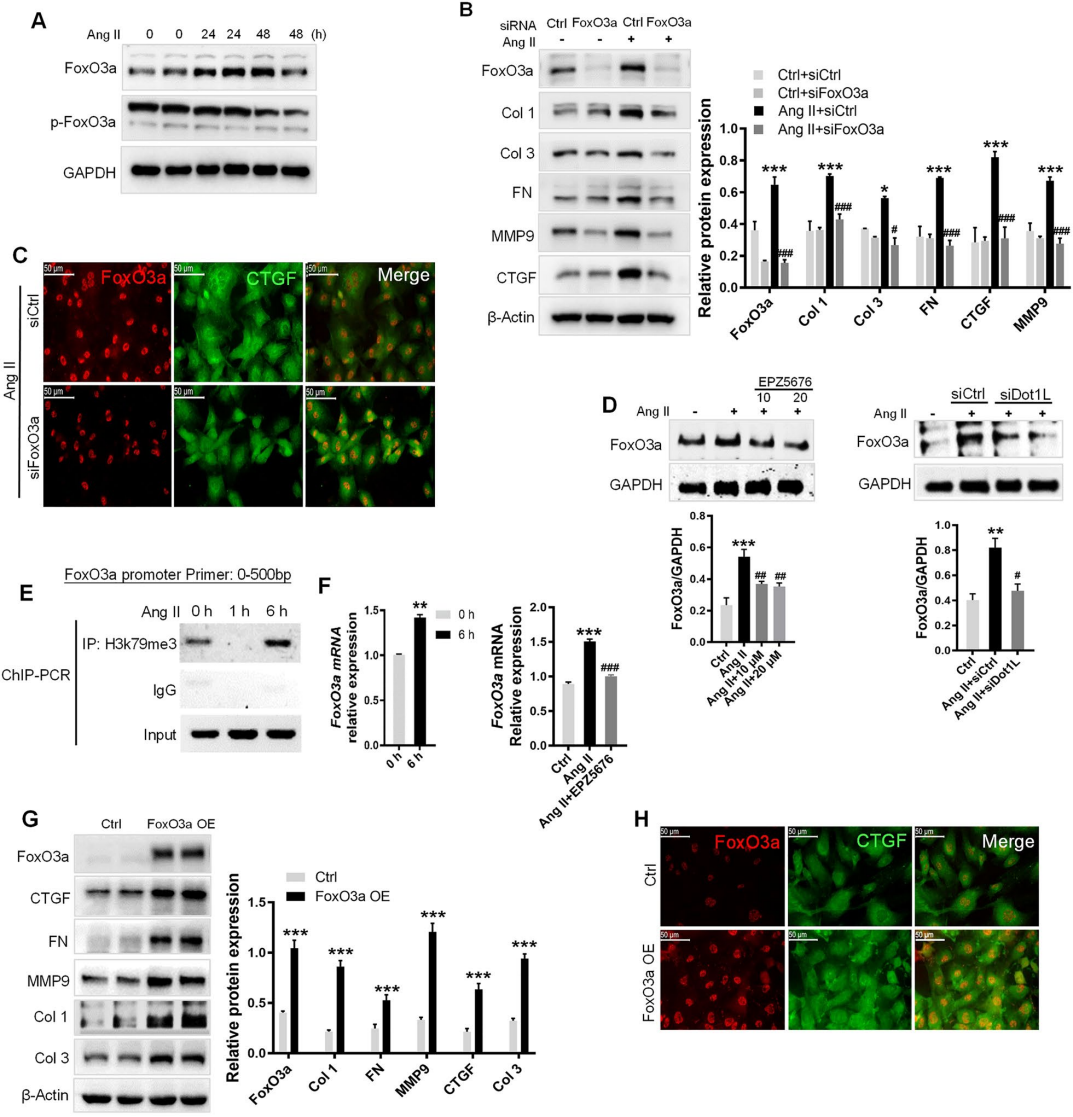
Dot1L directly binding to the promoter regions of FoxO3a promotes ECM deposition. **A** FoxO3a is upregulated in NRCFs treated with 2 μmol/L Ang II for 24 and 48 h. FoxO3a and its phosphorylation form were analyzed by western blot. **B**, **C** Knockdown of FoxO3a attenuates ECM deposition in Ang II-treated NRCFs. FoxO3a siRNA and control siRNA were transfected into NRCFs, followed by Ang II stimulation for 48 h. The protein expressions of FoxO3a, Col 1, Col 3, FN, MMP9 and CTGF were analyzed by western blot (B); Representative images of immunofluorescence staining of FoxO3a and CTGF in Ang II-stimulated NRCF (**C**). **D** NRCFs were pretreated with EPZ5676 (10, 20 μmol/L) for 4 h or transfected with siDot1L for 12 h, then incubated with 2 μmol/L Ang II for 48 h. FoxO3a was analyzed by western blot. **E** ChIP-PCR showing H3K79me3 gain in promoter of FoxO3a in Ang II-treated NRCFs. NRCFs treated with Ang II for 1 and 6 h were used for ChIP assay with antibody against H3K79me3. **F** mRNA level of FoxO3a was quantified by qRT-PCR after inhibition of Dot1L with EPZ5676. Above all data are represented as means ± SD. ANOVA, ^*^p < 0.05, ^**^p < 0.01, ^***^p < 0.001 versus Ctrl or Ctrl + siCtrl, ^#^*p* < 0.05, ^##^*p* < 0.01, ^###^*p* < 0.001 versus Ang II or Ang II + siCtrl, each acquired from three individual experiments. **G**, **H** Overexpression of FoxO3a promotes fibrosis response in NRCFs. NRCFs were transfected with lentivirus containing FoxO3a overexpression plasmid for 24 h, and then cultured with completed medium for 48 h. The protein expressions of FoxO3a, CTGF, FN, MMP9, Col 1 and Col 3 were analyzed by western blot. Data are represented as means ± SD. Student’s t-test, ^***^p < 0.001 versus Ctrl, each acquired from three individual experiments (**G**); immunofluorescence double staining of FoxO3a and CTGF (**H**)

We next investigated the underlying mechanism by which Dot1L promotes myocardial fibrosis. FoxO3a plays an important role in regulating several essential cellular functions. We found that an increased FoxO3a protein level by Ang II at time-dependent manner in CFs, while p-FoxO3a expressions were reduced (Fig. 7A). Meanwhile, we strikingly found that silencing FoxO3a expression exerted anti-fibrotic effect (Fig. 7B). Besides, immunofluorescence was performed to check synchronous change between FoxO3a and CTGF, which turned out to be consistent with the western blot results (Fig. 7C). Further, silencing or inhibiting Dot1L led to a decrease in FoxO3a protein level in Ang II-induced CFs (Fig. 7D). As Dot1L is the only methyltransferase targeting H3K79, the increase of H3K79me3 levels near the promoter of FoxO3a was validated by ChIP-PCR at Ang II-treated CFs (Fig. 7E). RT-qPCR validated the increased FoxO3a mRNA abundance upon Ang II treatment, instead, inhibiting Dot1L decreased FoxO3a transcription level (Fig. 7F). These data suggest that Dot1L transcriptionally activates FoxO3a via its H3K79 methylation dependent function.

Next, we sought to determine whether FoxO3a results in ECM deposition, we examined fibrosis marker expression through transfecting CFs cells with pCMV-FoxO3a. We observed that overexpression of FoxO3a (FoxO3a OE) in CFs could significantly cause consistently fibrosis marker expression such as CTGF, Col 1, Col 3, MMP9, and FN (Fig. 7G). Immunofluorescence analysis further revealed that overexpression of FoxO3a resulted in increased CTGF protein expression (Fig. 7H). These data suggest that FoxO3a positively regulates ECM deposition in CFs.

## Discussion

Excess activation and proliferation of CFs after MI greatly contribute to adverse cardiac remodeling and increase the risk of chronic heart failure. Dot1L mediated-methylation of histone H3K79 is symbolically presented in many actively transcribed genes [24]. In this study, we demonstrated that Dot1L could be a novel myofibroblasts activator, which upregulation in a mouse model of MI is an accomplice in myocardial fibrosis and consequent heart failure via FoxO3a-induced fibroblast activation and differentiation into collagen-producing myofibroblasts. In addition, we have also shown that the knockdown Dot1L or administration of specific inhibitor decreased myocardial fibrosis and improved heart function, and it ameliorated ventricular remodeling in mouse MI model. Thus, our study identifies Dot1L as a new player involved in myocardial fibrosis after MI injury.

Since the discovery of Dot1L as histone methyltransferase targeting on histone H3K79, great progress has been made in uncovering the role of this methylation modification in different physiological and pathological conditions, especially in the MLL-rearranged leukemias. Previous researches have demonstrated that a variety of epigenetic mechanisms was widely involved in cardiovascular diseases (CVDs) [25], as an important branch of epigenetic pathway, the role of Dot1L-mediated H3K79 methylation in CVDs is gradually revealed. Studies have shown that cardiac-specific conditional knockout of Dot1L in mice led to cardiac dilatation and postnatal lethality, confirmed that Dot1L activated key cardiac genes in cardiomyocyte differentiation and maturation by catalyzing different degrees of methylation on H3K79 [26, 27], indicating the important regulation of Dot1L in heart development. It’s also demonstrated that Dot1L was involved in atherosclerosis development through NF-κB pathway [28]. Our study revealed that Dot1L was a critical component of pro-fibrotic signaling after MI. Almost simultaneously, another work verified that Dot1L promoted the expression of spleen tyrosine kinase by increasing the H3K79me2 modification on its promoter and activated TGF-β1/Smad3-mediated myocardial injury[29]. Taken together, current researches have brought insight into the relevance of Dot1L to CVDs. There is a reasonable prospect that Dot1L would have enormous therapeutic potential in heart diseases coupled with in-depth knowledge of underlying mechanism of CVDs.

Our work also provided insights into the mechanism by which Dot1L inhibition affords cardioprotection following acute insults in MI model induced by LAD. In line with the previous reports, MI badly damages heart function [30, 31], which presents as attenuated cardiac contractile function along with significant structural remodeling, a significant decrease in ejection fraction and LV volume at both diastole and systole, indicating a progressive pathological remodeling of the myocardium in response to the MI insult. Further, we provided evidence that in mice with either systolic or diastolic dysfunction, higher level of Dot1L showed high commonality in severe cardiac interstitial fibrosis. Thus, efforts were made to test our hypothesis that upregulated cardiac Dot1L may mediate fibrosis and contribute to cardiac dysfunction. Fortunately, the following study illustrated that blockade of Dot1L in MI-induced mice cardiac remodeling alleviated fibrotic ECM deposition, and improved cardiac function. EPZ5676, a Dot1L specific inhibitor, treatment for 2 weeks post-MI alleviated cardiac interstitial fibrosis and the MI-induced increase in LV mass. Further, EF at 2 weeks after MI under treatment with EPZ5676 was better preserved than not, and same improvement on infarct scar thinning and infarct expansion were obtained. The latter is associated with decreased LV systolic function and increased infarct rupture at the infarct border zone [32]. In other words, treatment with EPZ5676 ameliorated the damage to LV systolic function and provided protection from infarct rupture, all of which contributed to better post-MI cardiac function. Meanwhile, Dot1L^+/-^ mice or knocking down of Dot1L by shRNA via a lentiviral vector limited cardiac fibrosis along with ameliorative cardiac function. The improvement of LV compliance and cardiac function could attribute to decreased LV fibrosis and cardiac remodeling.

Under the pathologically myocardial injury, fibroblasts activate and differentiate into myofibroblast, which display increased ability of proliferation, migration, and collagen production [33, 34]. Following an acute myocardial injury, CFs within the connective tissue convert to their activated form, which secrete elevated levels of ECM proteins to promote a profibrotic environment [35]. Cardiac fibrosis provokes a series of pathological changes including chamber dilatation, cardiomyocytes hypertrophy and apoptosis, and culminate in congestive heart failure [36]. Multiple signaling pathways are orchestrated in the cardiac fibrotic response. Among them, Ang II and TGF-β, have been demonstrated to be implicated in activation of CFs, fibroblast-to-myofibroblast transition and ECM deposition during the process of fibrosis [37]. We found that stimulation of normal fibroblasts with recombinant TGF-β or Ang II not only led to transformation into fibroblast phenotype, but also upregulated Dot1L expression. The upregulation of Dot1L in fibroblasts is thus consistent with fibrosis response. TGF-β or Ang II can induce serine phosphorylation of Smad2/3 and translocate into the nucleus to promote specific gene transcription [38]. We found Dot1L expression was mediated by Smad2/3 signaling and fibroblast activation was under the control of Dot1L. Blockade of Dot1L could significantly inhibit collagen deposition and myofibroblast differentiation.

Previous researches have established that Forkhead box O (FoxO) proteins, a transcription factor family, play a vital role in the regulation of cardiac fibroblast proliferation and differentiation [39]. Many proteins associated with fibrosis, such as Col 1, CTGF, and α-SMA can be used as biomarker for CFs differentiation. In skin fibroblasts, ultraviolet light increases Col 1 expression via FoxO1 activation, whereas FoxO1 down-regulation eliminates this effect [40]. Furthermore, FoxO1 is necessary for CTGF expression in HUVEC [41] or primary keratinocytes [42]. Our results showed that FoxO3a overexpression promoted Col 1, Col 3, FN, and CTGF expression. In corresponding, knockdown of FoxO3a inhibited the effects of Ang II on differentiation shown by multiply markers, indicating that FoxO3a contributing to the CFs differentiation induced by Ang II. Importantly, FoxO3a signaling pathway was mediated by Dot1L in Ang II-induced CFs, by a mechanism related to FoxO3a transcription regulation. Dot1L initiated H3K79me3 enrichment in the promoter of FoxO3a and consequently activated its transcription, consistent with Dot1L’s methyltransferase activity on H3K79. Taken together, results from this study suggest that Dot1L transcription regulates FoxO3a expression, which is crucial to the process of Ang II-induced CFs activation.

## Conclusions

In this study, we provided evidence that increased levels of Dot1L contributes to MI-induced myocardial fibrosis and dysfunction in mice. Additionally, we proved that in cardiac fibroblasts, the expression of Dot1L is upregulated under the stimulation of Ang II, cascading the activation of following profibrotic signaling. Thus, we proposed that myocardial Ang II signaling leads to upregulation of the Dot1L to facilitate differentiation from cardiac fibroblasts into collagen-producing myofibroblasts, which is vital to development of myocardial fibrosis and heart failure. Therefore, Dot1L may be a potential target for improving heart function following an ischemic insult.

## Materials and methods

### Reagents and antibodies

Transforming growth factor-β1 was obtained from ProteinTech (Proteintech, USA). Angiotensin II was obtained from Meilunbio (Meilun Biotechnology, Dalian, China). EPZ5676 (Dot1L inhibitor) was purchased from Selleckchem (Selleck, USA). Antibody against Dot1L (sc-390879) and CTGF (sc-365970) were obtained from Santa Cruz Biotechnology (Santa Cruz, CA); total H3 (4499), Smad2/3 (8685S) and p-Smad2/3 (8828S) were purchased from Cell Signaling Technology (Danvers, MA, USA). Antibody against CTGF (ab6992), H3K79me3 (ab2621), H3K79me1 (ab177183) and p-FoxO3a (ab154786) were purchased from Abcam; Col 3 (GB11023); MMP9 (GB12321-1), and α-SMA (GB13044) were purchased from Servicebio (Wuhan, China). Antibody against Dot1L (A11285), fibronectin (A7488), Col 1 (A5786), H3K79me2 (A2368) were purchased from ABclonal (Wuhan, China); FoxO3a (66,428-1-Ig) and GAPDH (60,004-1-Ig) were obtained from Proteintech (USA).

### Culture of primary adult and neonatal rat cardiac fibroblasts (CFs)

Adult Sprague–Dawley rats (130–150 g) and neonatal rat (1–3 days) were applied in the study. Briefly, the extracted ventricle from adult mice or neonatal rats was washed with cold PBS buffer and finely cut into pieces. Then the heart was digested in 0.25% trypsin with EDTA for 5 min at 37 °C with gently stirring all the time, which was repeated several times until the tissues disappeared. All digestive harvest was collected and centrifuged at 1000 rpm for 3 min. The cells were resuspended and plated for 2 h. Adherent cells were cardiac fibroblasts and cultured in DMEM completed medium (containing 10% fetal bovine serum, 1% penicillin and streptomycin), incubating at 37 °C in a humidified incubator with 5% CO_2_. CFs with passages between 1 and 3 were used for all studies.

### Western blot

The total protein extracted from cells or myocardial tissues using proper lysis solution were separated by SDS-PAGE, after which it was transferred to nitrocellulose membranes. Blocking in 5% skimmed milk to eliminate non-specific binding, then the membranes were incubated with appropriate primary antibodies overnight at 4 °C, followed by incubation with the speciesappropriate horseradish peroxidase (HRP)-labeled secondary antibody (Jackson ImmunoResearch Inc., USA). Protein-specific signals were detected using a Bio-Rad Imager (Bio-Rad, Hercules, CA, USA), and the relative expression levels were quantified by Alpha Imager (Alpha Innotech Corp, San Leandro, CA).

### Real time quantitative polymerase chain reaction (RT-qPCR)

After lysing with TRIzol Reagent (TaKaRa Biotechnology, Dalian, China), the total RNA of CFs was prepared by the general Trizol extraction protocol. Total RNA of each sample was reversely transcribed into cDNA and amplified using a PrimeScript 1st Strand cDNA Synthesis Kit (Takara) according to the manufacturer’s directions. Utilizing RT-qPCR, the fluorescence intensity was determined by iCycler iQ system (Bio-Rad, Hercules, CA, USA) for semi-qualification of FoxO3a expression level, with the housekeeping gene GAPDH used as control. Primer sequences were listed below: FoxO3a: 5 ´-TCCTGGCGGGCTTATGCAG-3 ´, 5 ´-GACATCATTGGGTCGTTGCG-3 ´. GAPDH: 5 ´-TTCAACGGCACAGTCAAGG-3 ´, 5 ´- CGGCATGTCAGATCCACAA-3 ´.

### Construction of FoxO3a overexpression plasmid

The full-length coding sequence of FoxO3a was obtained from UCSC Genome Browser. The primers and the restriction enzyme cutting site towards FoxO3a were get from Primer-BLAST of NCBI and Webcutter 2.0, respectively. Using the cDNA of rat genome as template, the target sequence was amplified by Q5 high-fidelity enzyme. Then, the product was cut and connected to lentivirus vector using corresponding enzymes. After transformed into Escherichia coli and cultured on LB medium, the single colony was picked and were send to Sanger sequencing. Effective colony was further amplified in LB and Hipure Plasmid EF Maxi kit (Magen, China) and was used for plasmid extraction according to manufacturer’s instructions.

### Lentivirus generation

Dot1L shRNA (shDot1L) and the negative control vector (shCtrl) were purchased from Public Protein/Plasmid Library. To obtain the lentivirus, the recombinant plasmid shDot1L, or FoxO3a overexpression plasmid (FoxO3a OE) and packaging vector pΔ8.2 and pVSVG were co-transfected into 293 T cells using transfection reagent lipofectamine 2000 (Invitrogen, USA). At 48 h and 72 h after transfection, the supernatant including lentivirus was collected and filtered by 0.45 μm filter, concentrated by ultracentrifugation (25 000 rpm, 4 °C, 2 h; Beckham SW 28 rotor) and then re-suspended. Lentivirus carrying a negative control vector was prepared to check the efficacy of lentivirus particle for transfection and control experiments.

### Small interfering RNA (siRNA) transfection

Dot1L siRNA (5′-GCAGAGGCUGUGUGACAAATT-3′; 5′-GCAGAAUCGUAUCCUCAAATT-3′; 5′-CCAAAGUCCCUGAGAGCAATT-3′), FoxO3a siRNA (5′-CCCAGAUCUACGAGUGGAUTT-3′), and control siRNA were synthesized by Genepharma (Shanghai, China). Smad2/3 siRNA was purchased from Santa Cruz. Transfection of siRNA was performed when cells plated on 6-well plates reached a confluence of 60–80% via Lipofectamine RNAiMAX. siRNA and Lipofectamine RNAiMAX were respectively diluted to proper concentration in Opti-MEM and then mixed and incubated at room temperature for 5 min, which was then added into the cells. After transfection for 24 h, the medium was replaced with serum-free DMEM medium and the efficiency of gene knockdown was examined by western blot at 72 h post-transfection.

### Animal studies

Permission was obtained from Institutional Animal Care and Use Committee (IACUC), Fudan University, China. All animals and the experimental protocol were completely in accord with the Animal Welfare Act Guide for Use and Care of Laboratory Animals.

C57BL/6 mice (25–27 g) going through operation were randomly divided into three groups: the sham control group, myocardial infarction group (MI), Dot1L inhibitor EPZ5676 group (MI + EPZ5676). Mice in the latter two group were subjected to permanent ligation of the left ascending artery (LAD) to induce myocardial infarction. In brief, mice were anesthetized by isoflurane with the assistance of endotracheal intubation and rodent ventilator. After opening the chest cavity and subsequently removing the pericardium, the beating heart would be accessible. The descending branch of the LAD coronary artery was located and occluded with a 7–0 prolene suture. Upon ligation, the lower left part of the left ventricle will instantly turn pale, indicating a successful operation. EPZ5676 at doses of 20 mg/kg/day was administered to mice by intraperitoneal injection until the day before sacrifice.

A recombinant lentivirus vector carrying shDot1L was generated to explore the effect of Dot1L knockdown in vivo. After LAD ligation, the 150 μL concentrated lentivirus carrying shDot1L or a negative control vector (shCtrl) was orthotopic injected around the edge of myocardial infarction area, respectively in shDot1L group and MI group. After 1 week, 150 μL concentrated lentivirus was supplemented via tail vein respectively. Ultrasonic cardiogram and other analysis were performed on the surviving animals after 2 weeks.

Dot1L heterozygous knockout (Dot1L^±^) mice were generated on a C57BL/6 background under CRISPR/Cas system, for the sake of homozygous deletion of Dot1L leaded to embryonic lethality. Genotyping was performed using genomic DNA extracted from tails.

### Echocardiography

Echocardiography was performed on spontaneously breathing mice as earlier described using VisualSonics Vevo 2100. Two-dimensional echocardiographic measurements were obtained. The ventricular dimensions (systolic and diastolic left ventricular internal diameter [LVIDs and LVIDd], as well as left ventricular end-diastolic and end-systolic volume [LVDD and LVSD]) were calculated and determined. Both ejection fraction (EF) and fractional shortening (FS) were calculated separately for left ventricular pump function evaluation.

### Histological evaluation and analysis of fibrosis

Mice were anesthetized by isoflurane and cervical dislocation after 14 days. The hearts were fixed in 4% Polyformaldehyde overnight and then were embedded in paraffin and stained with hematoxylin and eosin (H&E). To evaluate the histological change of fibrosis, the tissue sections were stained with Masson trichrome, according to the manufacturer’s instructions, which could show collagen deposition in blue. The percentage of infract area was defined by the ratio of fibrotic area with collagen deposition (blue) to the whole Masson’s trichrome-stained section measured by Image J. For Sirius Red staining, sections were deparaffinized and rehydrated through gradient concentration of alcohols to distilled water. The sections were stained with Sirius red for 60 min at room temperature, followed by staining with hematoxylin for 10 min, according by the manufacturer’s protocol. The collagen fibers including Col 1 and Col 3 were stained red, and muscle fibers as well as cytoplasm were stained yellow under light microscopy (Imager.M2, Zeiss, Germany).

### Immunohistochemistry (IHC)

10% normal goat serum was used to block the sections, while diluted hydrogen peroxide (1:10 dilution of 3% hydrogen peroxide) to quench endogenous peroxidases. Then, the sections were incubated with primary mouse antibodies (Col 3 or Dot1L) overnight at 4 °C. Subsequently, immunohistochemistry was performed according to the kit protocol of the Biotin-Streptavidin Horseradish Peroxidase (HRP) detection system, using biotin-labeled goat anti-rabbit immunoglobulin IgG and streptavidin/HRP (ThermoFisher). All images were captured using a microscope (Axio Scope.A1, Carl Zeiss Imaging Systems).

### Immunofluorescence

The cells seeded on glass coverslips going through corresponding treatment or tissue sections were fixed with 4% paraformaldehyde for 15 min. After permeabilization with 0.25% Triton X-100 for 10 min, the slides were blocked in 10% goat serum at least 30 min, and then incubated overnight with primary antibodies at 4 °C. Following incubation with appropriate secondary antibodies for 1.5 h at room temperature, the nuclei were stained with DAPI. All images were captured using a multi-channel fluorescence microscope (Zeiss).

### Chromatin immunoprecipitation PCR (ChIP-PCR)

Under the guidance of the standard cross-linking ChIP protocol, the ChIP assays between H3K79me3 and FoxO3a gene promoter were performed. In Brief, cells digested by trypsin were crosslinked with 1% formaldehyde for 15 min at room temperature. DNA were sheared by sonication to get fragments of 300–1000 bp size, which were then immunoprecipitated with specific antibodies, with the same amount of non-specific lgG as control. The immunoprecipitated complex was rinsed, and DNA was extracted and purified. ChIP DNA was analyzed by qPCR on the FoxO3a gene promoter. The primer used for ChIP-PCR was as follows: forward: TAGCCGGCTGCTTTATCCTA; reverse: ACAGTCGTGCTTCGCTTTTT.

### Statistical analysis

Utilizing the GraphPad Prism 7.0 software for statistical analysis, data were reported as mean values ± SD. Statistical significance between multiple groups were evaluated by one-way analysis of variance with Tukey’s test for post hoc comparisons. *p* < 0.05 was considered to be statistically significant.

## Supplementary Information

**Additional file 1:** **Fig. S1**. Dot1L expression is mediated by p-Smad3 in Ang II-induced CFs. (A) p-Smad3 is upregulated in NRCFs treated with 2 μmol/L Ang II for different times. Smad3 and its phosphorylation form were analyzed by western blot. (B) Smad2/3 knockdown attenuates Dot1L expression in Ang II treated NRCFs. Smad2/3 siRNA and control siRNA were transfected into NRCFs, followed by Ang II stimulation for 48 h. The protein expressions of Smad2/3, Dot1L, CTGF and Col 3 were analyzed by western blot. (C) Smad2/3 inhibition attenuates Dot1L expression in Ang II treated NRCFs. NRCFs were pretreated with Sis (0.5, 1, and 2.5 μmol/L) for 4 h and then co-incubated with 2 μmol/L Ang II for 48 h. Dot1L, Col 3, CTGF and MMP9 were analyzed by western blot. All data are presented as mean ± SD. ANOVA, ^***^p < 0.001 versus Ctrl or Ctrl+siCtrl, ^##^*p *< 0.01, ^###^*p *< 0.001 versus Ang II or Ang II+ siCtrl, each acquired from three individual experiments

## Data Availability

The datasets used and/or analysed during the current study are available from the corresponding author on reasonable request.

## Abbreviations

Ang II: Angiotensin II
TGF-β1: Transforming growth factor-β1
CFs: Cardiac fibroblasts
NRCFs: Neonatal rat cardiac fibroblasts
Col 1: Collagen I
Col 3: Collagen III
CTGF: Connective tissue growth factor
Dot1L: Disruptor of telomeric silencing 1-like
ECM: Extracellular matrix
FMT: Fibroblast-to-myofibroblast transition
FN: Fibronectin
FoxO: Forkhead box O
LAD: Left ascending artery
MI: Myocardial infarction
MMPs: Matrix metalloproteinase
α-SMA: α-Smooth muscle actin
