# Supplementary material for: Inhibition of the cardiac fibroblast-enriched histone methyltransferase Dot1L prevents cardiac fibrosis and cardiac dysfunction
Source: Cell Biosci. 2022 Aug 19;12:134. doi: 10.1186/s13578-022-00877-5 (PMC9392317; doi:10.1186/s13578-022-00877-5)
Supplement: Supplementary file 1 — Additional file 1: Fig. S1. Dot1L expression is mediated by p-Smad3 in Ang II-induced CFs. (A) p-Smad3 is upregulated in NRCFs treated with 2 μmol/L Ang II for different times. Smad3 and its phosphorylation form were analyzed by western blot. (B) Smad2/3 knockdown attenuates Dot1L expression in Ang II treated NRCFs. Smad2/3 siRNA and control siRNA were transfected into NRCFs, followed by Ang II stimulation for 48 h. The protein expressions of Smad2/3, Dot1L, CTGF and Col 3 were analyzed by western blot. (C) Smad2/3 inhibition attenuates Dot1L expression in Ang II treated NRCFs. NRCFs were pretreated with Sis (0.5, 1, and 2.5 μmol/L) for 4 h and then co-incubated with 2 μmol/L Ang II for 48 h. Dot1L, Col 3, CTGF and MMP9 were analyzed by western blot. All data are presented as mean ± SD. ANOVA, ***p < 0.001 versus Ctrl or Ctrl+siCtrl, ##p < 0.01, ###p < 0.001 versus Ang II or Ang II+ siCtrl, each acquired from three individual experiments [file 13578_2022_877_MOESM1_ESM.docx]

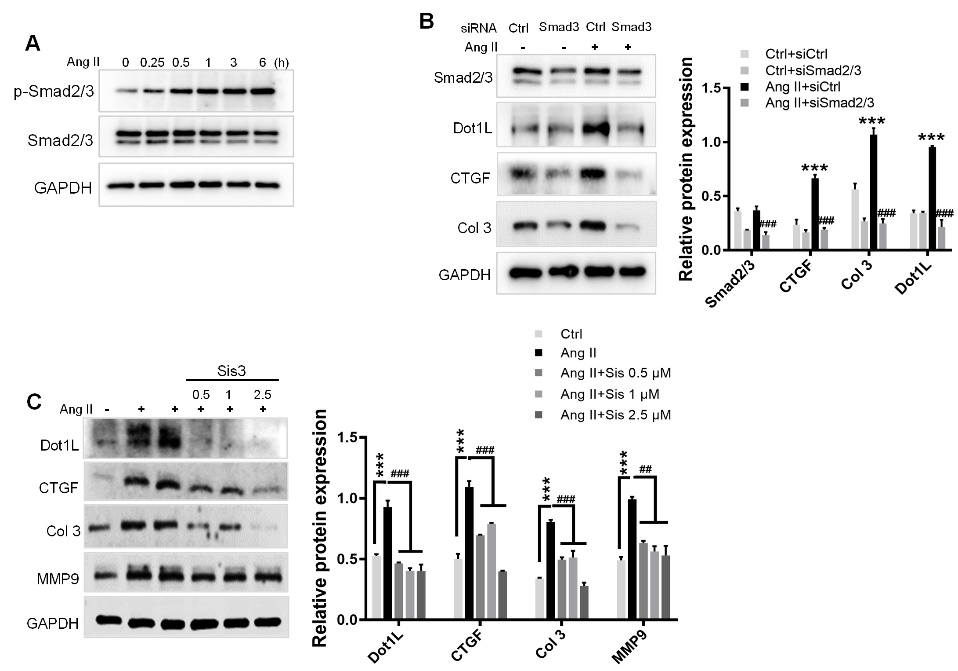


**Fig. S1 Dot1L expression is mediated by p-Smad3 in Ang II-induced CFs.** (A) p-Smad3 is upregulated in NRCFs treated with 2 μmol/L Ang II for different times. Smad3 and its phosphorylation form were analyzed by western blot. (B) Smad2/3 knockdown attenuates Dot1L expression in Ang II treated NRCFs. Smad2/3 siRNA and control siRNA were transfected into NRCFs, followed by Ang II stimulation for 48 h. The protein expressions of Smad2/3, Dot1L, CTGF and Col 3 were analyzed by western blot. (C) Smad2/3 inhibition attenuates Dot1L expression in Ang II treated NRCFs. NRCFs were pretreated with Sis (0.5, 1, and 2.5 μmol/L) for 4 h and then co-incubated with 2 μmol/L Ang II for 48 h. Dot1L, Col 3, CTGF and MMP9 were analyzed by western blot. All data are presented as mean ± SD. ANOVA, ^***^*p* < 0.001 versus Ctrl or Ctrl+siCtrl, ^##^*p* < 0.01, ^###^*p* < 0.001 versus Ang II or Ang II+ siCtrl, each acquired from three individual experiments.
